# Efficacy and Mechanism of Buyang Huanwu Decoction in Patients With Ischemic Heart Failure: A Randomized, Double-Blind, Placebo-Controlled Trial Combined With Proteomic Analysis

**DOI:** 10.3389/fphar.2022.831208

**Published:** 2022-03-18

**Authors:** Mingjun Zhu, Jingjing Wei, Ying Li, Yongxia Wang, Junguo Ren, Bin Li, Bo Ma, Xinlu Wang, Lijie Qiao, Cheng Zhou, Jianxun Liu

**Affiliations:** ^1^ First Affiliated Hospital of Henan University of CM, Zhengzhou, China; ^2^ Henan University of Chinese Medicine, Zhengzhou, China; ^3^ Beijing Key Laboratory of Pharmacology of Chinese Materia Region, Institute of Basic Medical Sciences, Xiyuan Hospital, China Academy of Chinese Medical Sciences, National Clinical Research Center of Cardiovascular Disease of Traditional Chinese Medicine, Beijing, China

**Keywords:** ischemic heart failure, Buyang Huanwu decoction, randomized controlled trial, proteomics, energy metabolism, coagulation function

## Abstract

**Objective:** Buyang Huanwu Decoction (BYHW), a famous herbal prescription in traditional Chinese medicine (TCM), has been used for 200 years for treating ischemic heart failure (IHF). This study aims to assess the efficacy and safety of BYHW combined with guideline-guided pharmacotherapy in patients with IHF and explore the biological mechanism by which BYHW exerts its efficacy.

**Methods:** In the multicenter, double-blind, randomized controlled trial, a total of 80 patients with IHF were randomized to receive BYHW or placebo for 3 months. The primary efficacy endpoints were New York Heart Association (NYHA) classification, TCM syndrome scores, N-terminal pro-B-type natriuretic peptide (NT-ProBNP), whereas the mechanism exploration endpoints included energy metabolism parameters and coagulation function parameters. In addition, we performed the proteomic study of the serum of patients after treatment by label-free quantification technology to verify the candidate target proteins and pathways.

**Results:** After 3 months of treatment, the NYHA classification, TCM syndrome scores, and the percentage of subjects with at least 30% reduction in NT-ProBNP were significantly improved in the BYHW group, compared with the control group (*p* < 0.05); BYHW treatment also significantly regulated blood glucose, blood lipid levels, ameliorated energy metabolism and improved coagulation function parameters. There were no significant differences in safety endpoints between the two groups. In addition, we obtained 56 differentially expressed proteins by proteomics, including 20 upregulated proteins and 36 downregulated proteins. Bioinformatic analysis revealed the mechanism of BYHW treatment was significantly related to complement and coagulation cascades, cholesterol metabolism, NF-kappa B signaling pathway, PI3K-Akt signaling pathway, and metabolic pathways. Among these differentially regulated proteins, fibrinogen gamma (FGG), fibrinogen beta (FGB), Carboxypeptidase B2 (CPB2), Coagulation factor XIII A (F13A1), Intercellular adhesion molecule1 (ICAM1), Apolipoprotein C-II(APOC2), Apolipoprotein C-I(APOC1), and CD44 were found to be signature proteins associated with the efficacy of BYHW against IHF.

**Conclusion:** BYHW treatment can further improve cardiac dysfunction and clinical symptoms in IHF based on standard therapy without apparent adverse effects. Additionally, BYHW may play a therapeutic role in IHF by improving energy metabolism and regulating coagulation function through multiple targets and pathways.

**Clinical Trial Registration:** clinicaltrials.gov, identifier NCT02875639

## 1 Introduction

Ischemic heart failure (IHF) remains a significant cause of morbidity and mortality worldwide. More than 37.7 million patients suffer from HF worldwide, and about 70% of HF can trace back to ischemic heart disease (Ziaeian and Fonarow, 2016; Elgendy et al., 2019). More than 8.9 million patients with HF have been in China, and coronary heart disease has become the dominant primary disease in Chinese patients (Ma et al., 2020). With the development of early reperfusion strategies, including coronary stent implantation and drug therapy, the short-term mortality after acute myocardial infarction has been significantly reduced. However, the prevalence of IHF has been increasing year by year. After myocardial infarction, about 40%–56% of patients will have decreased cardiac function, about 25%–33% of patients will develop HF (Minicucci et al., 2011). Therefore, it is essential to effectively improve patients’ clinical symptoms and quality of life with IHF to prevent or reverse cardiac remodeling.

In recent years, clinical randomized controlled trials have confirmed the safety and effectiveness of traditional Chinese medicine (TCM) in treating IHF (Li et al., 2013). According to the TCM theory, the leading causes of IHF are deficiency of heart-qi and blood stasis. The theory further defines the Buyang Huanwu Decoction (BYHW) as a classic prescription for supplementing heart-qi and activating blood circulation. To specify, BYHW consists of seven commonly used Chinese herbal medicines, all registered in the Chinese Pharmacopoeia:

Astragali Radix (*Astragalus membranaceus* (Fisch.) Bge. var. mongholicus (Bge.), Hsiao or *Astragalus membranaceus* (Fisch.) Bge., Leguminosae), Paeoniae Radix Rubra (*Paeonia lactiflora* PalL or Paeonia veitchii Lynch, Ranunculaceae), Radix Angelicae Sinensis (*Angelica sinensis* (Oliv.) Diels., Umbelliferae), Rhizoma Chuanxiong (*Ligusticum chuanxiong* Hort., Umbelliferae), Flos Carthami (*Carthamus tinctorius* L., Feverfew), Pheretima (*Pheretima aspergillum* (E. Perrier) or Pheretima vulgaris Chen or *Pheretima guillelmi* (Michaelsen) or *Pheretima pectinifera* Mkhaeken, Megascolecidae), and Persicae Semen (*Prunus persica* (L) Batsch or *Prunus davidiana* (Carr.) Franch., Rosaceae).

The Preparation and assay methods of BYHW followed the guidelines of the Pharmaceutical standards of the Ministry of Health of the People’s Republic of China. By using the method of liquid chromatography tandem-mass spectrometry (LC-MS/MS), we identified 11 compounds from the BYHW granules. (Please see Supplementary Material for more information). Previous studies have shown that BYHW can regulate lipid metabolism, improve hemorheology, enhance plaque stability, and protect cardiac function (Wang et al., 2011; Chen et al., 2021). Although numerous clinical trials reported the efficacy of BYHW in treating IHF (Wu et al., 2021), the underlying mechanism of BYHW in treating IHF remains unclear.

Proteomics is a discipline based on mass spectrometry technology. The research thinking of proteomics is very similar to the holistic and multi-targeted view of TCM. By characterizing Disease symptoms at the molecular level by detecting the protein expression and modification of clinical samples, the discipline offers a new approach and theoretical support for Chinese medicine’s clinical diagnosis and treatment (Wei et al., 2019).

In this paper, we ensure the clinical trial meets the standards of randomized, multicenter, double-blind, and placebo-controlled. Our objective is to evaluate the efficacy and safety of BYHW combined under the guidelines for IHF pharmacotherapy and to reveal the biological mechanism by label-free quantification proteomics.

## 2 Methods

### 2.1 Trial Design

A multicenter, randomized, double-blind, placebo-controlled, parallel-group clinical study was conducted in China from June 2016 to December 2019 to reveal the therapeutic mechanism of BYHW in the treatment of IHF (qi deficiency and blood stasis syndrome). The trial consisted of 80 subjects from three clinical trial sites in Henan Province, China, including The First Affiliated Hospital of Henan University of Chinese Medicine, Henan Province Hospital of TCM, and Zhengzhou Hospital of TCM. This study observed the World Medical Association’s Declaration of Helsinki and the regulations and guidelines of China on Good Clinical Practice. The trail obtained ethical approval from the Ethics Committee of the First Affiliated Hospital of Henan University of Chinese Medicine (Approval number: 2015HL-045-01). This study has been registered with the US Clinical Trials Registry (https://clinicaltrials.gov; identifier NCT02875639).

### 2.2 Participants

#### 2.2.1 Inclusion Criteria

1) Age 40 to 75; 2) Patients with ischemic heart failure: left ventricular ejection fraction (LVEF) less than or equal to 45% measured by echocardiography in modified Simpson method; History of myocardial infarction with or without percutaneous coronary intervention or coronary artery bypass grafting; Coronary angiography or coronary computed tomography angiography shows ≥50% stenosis in at least one main coronary artery with or without revascularization, which the researcher thinks is closely related to HF; With or without dyspnea, fatigue and fluid retention (edema); 3) Qi deficiency and blood stasis syndrome (Ren et al., 2012); 4) New York Heart Association (NYHA) Class II to III; 5) Submitted informed consent.

#### 2.2.2 Exclusion Criteria

1) Combine the pulmonary embolism, or acute coronary syndrome, or acute cerebrovascular disease; 2) Combine other heart diseases: valvular heart disease, dilated cardiomyopathy, hypertension heart disease, pulmonary heart disease, congenital heart disease; 3) Severe hepatic and renal dysfunction, malnutrition, malignant tumor; 4) Psychosis and drug abuse; 5) Absolute contraindications to TCM; 6) Being pregnant, planning for pregnancy or breastfeeding.

### 2.3 Interventions

Before registration, the trial provided informed consent to all eligible patients in written form. Subsequently, all registered patients underwent a screening process for 1 week. During this period, the patients received western medicine treatments according to their clinical conditions under the Guidelines for Heart Failure established by the American College of Cardiology Foundation/American Heart Association or the Chinese Medical Association (Yancy et al., 2013; Cardiovascular Society Heart Failure Group of Chinese Medical Association, 2018). Western medicine treatments included diuretics, angiotensin-converting enzyme inhibitors or angiotensin receptor blockers, β-receptor blockers, aldosterone receptor antagonists, digoxin, and vasodilators. After rigorous screening, the trial randomly assigned stable subjects to either the experimental or control group. In addition to their standardized western medicine, patients assigned to the experimental or control group took BYHW or placebo, respectively, for 3 months. The composition of BYHW was 60 g of Astragali Radix, 15 g of Paeoniae Radix Rubra, 20 g of Radix Angelicae Sinensis, 12 g of Rhizoma Chuanxiong, 12 g of Pheretima, 12 g of Flos Carthami, and 12 g of Persicae Semen, totaling 143 g of the crude drug. Its formula granules (19.4 g) were prepared in the same proportion by Sanjiu Medical & Pharmaceutical Co., Ltd. (Shenzhen, China), with a TCM simulant as the placebo. They were similar to each other in strength, appearance, and odor. The investigational drug (granules in 19.4 g) was orally given to the patients twice a day, 1 pack per dosing. The trial did not apply additional Chinese herbs other than BYHW or placebo. The investigational drug was taken routinely for 3 months unless not allowed due to specific clinical circumstances. Patients were free to withdraw from the trial when necessary.

### 2.4 Outcomes

The primary efficacy endpoints included NYHA classification, TCM syndrome scores, NT-ProBNP. The efficacy standard was developed under the Principles for Clinical Research of New Drugs of Traditional Chinese Medicine in the Treatment of Heart Failure (Wang et al., 2019). The secondary efficacy endpoints included a 6-minute walk distance (6MWD) and LVEF. Mechanism exploration endpoints included energy metabolism indexes [Adenosine Diphosphate (ADP)/Adenosine Triphosphate (ATP) Ratio; Glucose (GLU); total cholesterol (TC); triglyceride (TG); high-density lipoprotein cholesterol (HDL-C); low-density lipoprotein cholesterol (LDL-C)], and coagulation function parameters [Prothrombin time (PT); Activation time of partial thromboplastin (APTT); Fibrinogen (FIB); and Thrombin Time (TT)]. The safety endpoints included tests of blood, urine, Electrolytes (K, Na, Cl), Hepatic and renal function. The above endpoints were observed and recorded once at months 0 and 3, respectively, during the treatment period. The adverse events (AEs)/adverse drug reactions (ADRs) record form was truthfully completed during the trial.

### 2.5 Sample Size

The sample used to explore the efficacy and mechanism was small in terms of size. According to expert opinions, the minimum sample size required for enrollment subjects in this study was 30 cases in each group. Considering a dropout rate of about 20% in each group, the sample size in each group was chosen to be 40 cases. Therefore, we enrolled 80 patients in this study and subsequently randomized the patients in a 1:1 ratio to the BYHW and control groups.

### 2.6 Randomization

The randomization procedure was designed by the Data Manager, Xiyuan Hospital of China Academy of Chinese Medical Sciences, using SAS statistical software. Block randomization was performed using the PROC PLAN process (block length of 10, in 8 blocks). The resulting random numbers were hidden in sealed opaque envelopes by investigators not involved in the recruitment. The therapists were responsible for sequentially opening the randomized envelopes and allocating the subjects accordingly. Patients, participants, and the study statistician were all blinded to treatment allocation. Each group of TCM investigational drug and TCM simulants passed the drug inspection and were entrusted to China Resources Sanjiu Medical & Pharmaceutical Co., Ltd., for manufacturing. An independent drug administrator was responsible for the delivery and distribution of the drugs used in the study.

### 2.7 Proteomics Analysis

#### 2.7.1 Preparation of Serum Samples

The study randomly chose 30 patients from each group among all subjects who completed the trial. The same amount of serum from 10 participants, five male and five female, was mixed, and the operation was repeated three times. After freezing, the serum was thawed at room temperature. The serum underwent shaking for 1 min, with 50 μl taken as a sample to be transferred into the EP tube. The high-abundance protein removal kit was transferred to a 10 kDa ultrafiltration tube and concentrated to 50 μl, which was replaced into an 8 mol L^−1^ solution system. The final concentration of 50 mmol L^−1^ 1,4-DTT was used for reduction, alkylation (dark) using a final concentration of 100 mmol L^−1^ IAA, and subsequent urea displacement by 50 mmol L^−1^ NH_4_CO_3_ solutions, followed by incubation at 95°C for 10 min, enzymatic digestion (37°C, 16 h) using trypsin added at 1:50 mass ratio of enzyme to substrate, terminated by adding 0.1% FA. Protein concentration was identified by the bicinchoninic acid method.

#### 2.7.2 Main Instrument and Reagents

The instrument and reagents involved in the study were purchased from the following organizations:

Easy nLC 1,000 nA Upgrade Liquid Chromatograph: Thermo Fisher, United States. Q Exactive Plus (QE Plus): Thermo Fisher, United States. Chromatographic water: Hangzhou Wahaha Group Co., Ltd., China. Urea: Affymetrix, United States. Sequencing grade Trypsin: Promega, United States. Formic Acid (FA), Dithiothreitol (DTT), and acetonitrile (ACN): Thermo Fisher, United States. Iodoacetamide (IAA): GE Healthcare, United States. Ammonium Bicarbonate: Amresco, United States.

#### 2.7.3 LC-MS/MS Analysis

The samples went through an Easy nLC 1,000 nL liquid chromatography. The flow phase A was 0.1% FA and double steam water, the flow phase B was 0.1% FA and ACN, gradient elution: 0∼3 min, 3%–6% B; 3–78 min, 6%–22% B; 78–79 min, 22%–100% B; 79∼107 min, 100% B. The flow rate is 400 nL min^−1^. Mass spectrometry was performed on a QE Plus mass spectrometer in data-dependent acquisition mode. The resolution of the first-order mass spectrum was set to 70,000, the scan range was m/z 300-1,800, the automatic gain control value was 3 × 106, and the injection time was 50 ms. The resolution of the secondary mass spectrum was set to 17,500, the automatic gain control value was 1 × 10^5^, the injection time was 45 ms, and the impact energy was 27 NCE. The standard for the second collection was set to the top 20 most intense ions as parent ions before Orbitrap detection. Each sample was repeated three times by mass spectrometry.

#### 2.7.4 Protein Identification and Quantitative Analysis

The RAW data obtained by mass spectrometry were imported into Proteome Discoverer software for protein identification. The protein database was from UniProt (http://www.uniprot.org/). The parameters were established as follows:

Confidence of peptide: high
Maximum number of protein leakage sites: 2
Length range of peptide: 6–144 amino acids
Mass deviation of parent ion: ± 10
Mass deviation of fragment ion: 0.02
Fixed modification: cysteine iodoacetamide
Variable modification: methionine oxidation and N-acetylation
False discovery rate of peptide library search: 1%

The RAW data was evaluated by Maxquant software via quantitative analysis, and the protein database was the same as above. Below were the standards of the parameters:

Maximum number of protein leakage sites: 2
Fixed modification: cysteine iodoacetamide
Variable modification: methionine oxidation and N-acetylation
Mass deviation of precursor ions: ± 20
Fragment ion mass deviation: 0.02
Minimum detectable peptide: 7 amino acids
False discovery rate of peptide library search: 1%.

#### 2.7.5 Bioinformatics Analysis

The study identified proteins with a statistically significant and fold change >1.5 as differentially expressed proteins. Function and pathway enrichment analyses were performed by searching the differentially expressed proteins against the Gene Ontology (GO) (http://www.geneontology.org/) and Kyoto Encyclopedia of Genes and Genomes (KEGG) (http://www.genome.jp/kegg/pathway.html) database. For protein-protein interaction analysis, the study referred to the Search Tool for the Retrieval of Interacting Genes/Proteins (STRING) (https://string-db.org/).

### 2.8 Statistical Analysis

All statistical analyses were performed using SAS statistical software version 9.2 (SAS Institute). The baseline characteristics of all subjects who received randomized treatment were analyzed according to the Full Analysis Set (FAS) principle. The data of subjects who completed all trials were examined based on the Per Protocol Set (PPS) principle. Continuous variables were presented as the mean ± standard deviation (SD). Under the condition of normal distribution and equal variance, the t-test was applied to compare the two groups while the Wilcoxon rank-sum test was used as an alternative; binary variables expressed as frequency or percentage were estimated using chi-square or Fisher exact test. *p* < 0.05 was considered statistically significant.

## 3 Results

### 3.1 Clinical Characteristics

**FIGURE 1:**
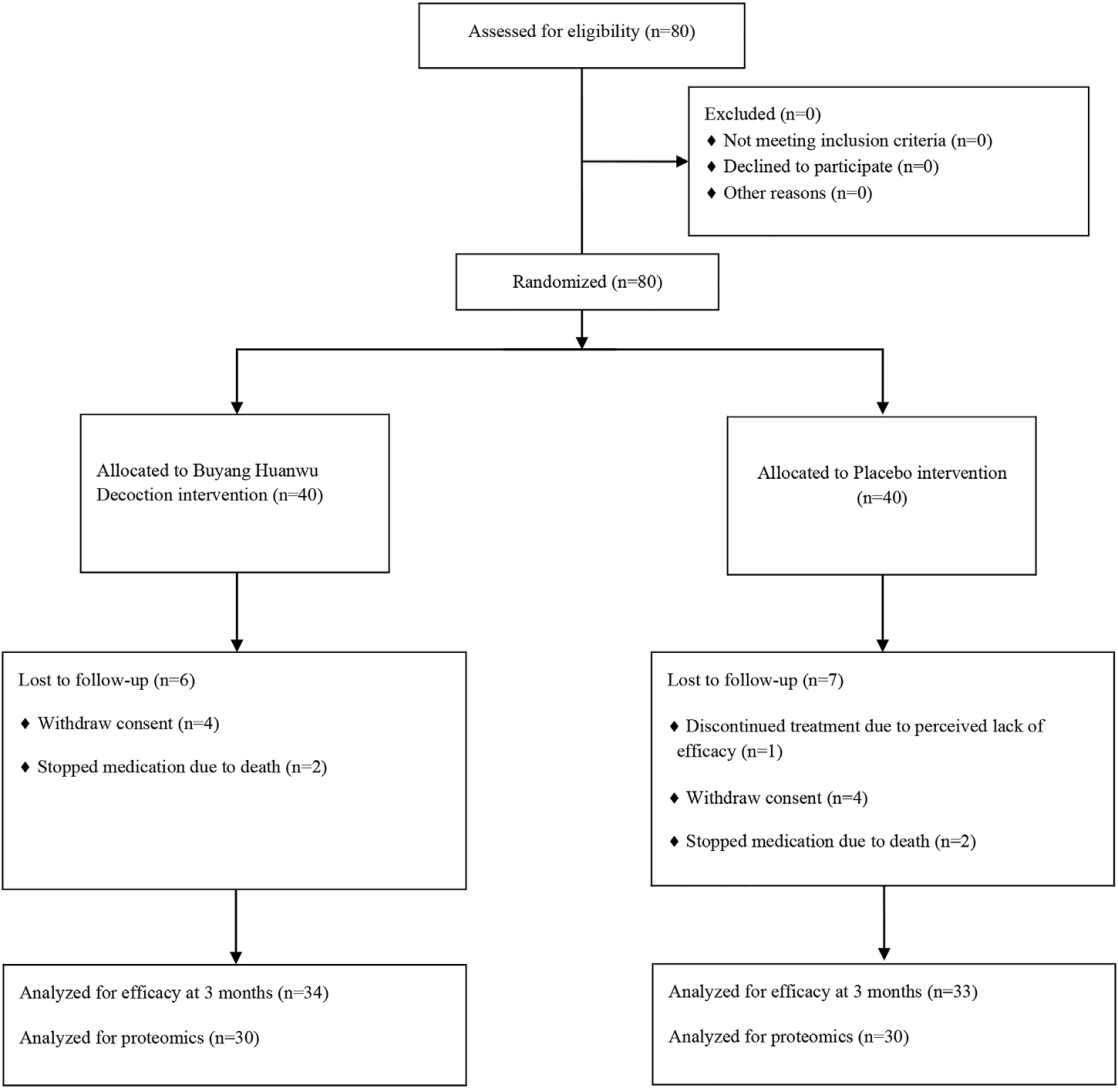
Flow diagram of patient selection and trial design.

The study contained, in total, 80 patients with IHF. After the strict screening process, 80 patients with IHF were finally included and randomized to the control and BYHW groups (Figure 1). Among the participants, 67 patients completed the 3-month treatment period. During the trial, a total of 9 subjects dropped out (4 in the BYHW group and 5 in the control group), causing an 11.25% dropout rate. The main reason for dropout was that the subjects were unwilling or unable to continue the clinical trial and voluntarily requested to withdraw. In addition, 4 patients died due to their disease worsening (2 in the BYHW group and 2 in the control group). There was no significant difference in demographics, vital signs, medical history, background drug information, NYHA classification, and TCM syndrome scores between the two groups (*p* > 0.05; Table 1).

**TABLE 1:** Baseline characteristics of participants between the BYHW and control groups.

Characteristic	BYHW (*n* = 40)	Control (*n* = 40)	*p* value
Basic information			
Age (years)	64.68 ± 7.89	64.93 ± 8.13	0.889
Male n (%)	27 (67.50%)	26 (65.00%)	0.813
Female n (%)	13 (32.50%)	14 (35.00%)	
BMI (kg/cm^2^)	24.72 ± 3.70	24.27 ± 3.60	0.584
SBP (mmHg)	129.40 ± 17.35	127.80 ± 17.70	0.684
DBP (mmHg)	80.22 ± 9.94	77.50 ± 11.15	0.252
Heart rate (bpm)	69.65 ± 7.75	66.72 ± 7.74	0.097
Respiratory rate (times/min)	18.50 ± 1.15	18.63 ± 1.24	0.628
Medical history n (%)			
History of hypertension	13 (44.80%)	20 (48.80%)	0.112
History of diabetes	8 (27.60%)	9 (22.00%)	0.785
Hyperlipidemia	5 (17.20%)	7 (17.10%)	0.531
Treatment n (%)			
Antiplatelet	37 (92.50)	35 (87.50%)	0.456
Beta-blockers	19 (47.50%)	28 (70.00%)	0.041
ACEI/ARB	17 (42.5%)	12 (30.00%)	0.271
Calcium antagonists	3 (7.50%)	7 (17.50%)	0.176
Statins	32 (80.00%)	33 (82.50%)	0.775
Diuretics	13 (32.50%)	10 (25.00%)	0.459
Aldosterone receptor antagonist	11 (27.50%)	12 (30.00%)	0.812
Nitric acid lipid	9 (22.50%)	11 (27.50%)	0.606
Digoxin	8 (20.00%)	4 (10.00%)	0.21
Clinical index			
TCM syndrome score	14.65 ± 3.16	15.75 ± 4.28	0.1948
NYHA classification			
II n (%)	25 (62.5%)	25 (62.5%)	1
III n (%)	15 (37.5%)	15 (37.5%)	

BYHW, Buyang Huanwu decoction; BMI, body mass index; SBP, systolic blood pressure; DBP, diastolic blood pressure; ACEI, angiotensin converting enzyme inhibitors; ARB, angiotensin receptor blocker; TCM, traditional Chinese medicine; NYHA, New York Heart Association.

### 3.2 Comparison of Efficacy Endpoints Between the BYHW and Control Groups

After 3 months, BYHW treatment significantly improved NYHA classification by 55.88% compared with the 36.36% increase observed in the control group (*p* = 0.038); Similarly, BYHW treatment showed high efficacy (70.59%) in improving TCM syndrome scores compared with the 48.48% increase observed in the control group (*p* = 0.036) (See details in Table 2).

**TABLE 2:** Comparison of NYHA classification and TCM syndrome scores efficiency between BYHW and control groups after 3 months of treatment.

	Excellent	Valid	Invalid	Worsened	Effective rate (%)	*p* value
NYHA classification efficiency						
BYHW (*n* = 34)	16	3	13	2	55.88	0.038
Control (*n* = 33)	12	0	12	9	36.36	
TCM syndrome scores efficiency						
BYHW (*n* = 34)	6	18	10	0	70.59	0.036
Control (*n* = 33)	9	7	16	1	48.48	

As shown in Table 3, there was no significant difference in NT-ProBNP, 6 MWD, and LVEF between the two groups before and after treatment (*p* > 0.05); compared with the same group before treatment, BYHW treatment significantly improved NT-ProBNP, 6 MWD, and LVEF as the control group (*p* < 0.01). In addition, 76.00% of subjects from the BYHW group had a decrease in NT-ProBNP of at least 30% compared with 48.10% of subjects from the control group (*p* < 0.05).

**TABLE 3:** Comparison of change in NT-ProBNP, 6 MWD and LVEF between BYHW and control groups after 3 months of treatment.

	BYHW (*n* = 34)	Control (*n* = 33)	*p* value
NT-ProBNP (pg/ml)			
Before treatment	2017.67 ± 3,094.69	1954.26 ± 2,690.37	0.904
After treatment	1,211.03 ± 1,663.36**	1931.23 ± 4,307.86*	0.406
Proportion of patients with a reduction in NT-proBNP at least 30% (%)	76.00%	48.10%	0.039
LVEF (%)			
Before treatment	41.09 ± 8.05	42.22 ± 7.85	0.563
After treatment	49.12 ± 9.82**	47.62 ± 10.25**	0.543
6 MWD (m)			
Before treatment	372.10 ± 85.24	363.82 ± 97.72	0.712
After treatment	435.91 ± 68.62**	399.88 ± 126.03*	0.149

NT-proBNP, N-terminal pro-B-type natriuretic peptide; LVEF, left ventricular ejection fraction; 6 MWD, 6-minute walking distance; compared with the same group before treatment, *p < 0.05, **p < 0.01.

### 3.3 Comparison of Mechanism Exploration Endpoints Between the Buyang Huanwu Decoction and Control Groups

From the data in Table 4, there was no significant difference in ADP/ATP Ratio, TC, TG, HDL-C, and LDL-C between the two groups before and after treatment (*p* > 0.05). When compared with the same group before treatment, there was a significant improvement in ADP/ATP Ratio, TC and LDL-C in the BYHW group (*p* < 0.05), but hardly any difference in the control group (*p* > 0.05). In addition, GLU varied distinctly between the two groups before and after treatment (*p* < 0.05).

**TABLE 4:** Comparison of change in energy metabolism indexes between BYHW and control group after 3 months of treatment.

	BYHW (*n* = 34)	Control (*n* = 33)	*p* value
ADP/ATP Ratio			
Before treatment	1.43 ± 2.80	1.00 ± 0.69	0.395
After treatment	0.76 ± 0.47*	0.80 ± 0.76	0.796
GLU (mmol/L)			
Before treatment	7.99 ± 4.00	6.40 ± 2.12	0.046
After treatment	6.42 ± 1.90**	5.46 ± 1.79**	0.039
TC (mmol/L)			
Before treatment	4.01 ± 1.06	4.34 ± 1.21	0.24
After treatment	3.55 ± 0.81*	3.98 ± 0.92	0.05
TG (mmol/L)			
Before treatment	1.66 ± 1.20	1.70 ± 0.92	0.891
After treatment	1.51 ± 0.63	1.76 ± 1.22	0.294
HDL-C (mmol/L)			
Before treatment	1.18 ± 0.29	1.11 ± 0.21	0.264
After treatment	1.29 ± 0.29	1.20 ± 0.22	0.128
LDL-C (mmol/L)			
Before treatment	2.50 ± 0.75	2.57 ± 0.91	0.74
After treatment	2.09 ± 0.68**	2.42 ± 0.73	0.064

ADP, adenosine diphosphate; ATP, adenosine triphosphate; GLU, glucose; TC, total cholesterol; TG, triglyceride; LDL-C, low density lipoprotein cholesterol; HDL-C, high density lipoprotein cholesterol, *p < 0.05, **p < 0.01.

As shown in Table 5, there was no prominent difference in PT, APTT, FIB, and TT at baseline between the two groups (*p* > 0.05). After 3 months, BYHW treatment dramatically improved TT compared with the control group (*p* < 0.05); compared with the same group before treatment, BYHW treatment notably improved APTT, FIB, and TT (*p* < 0.05).

**TABLE 5:** Comparison of change in coagulation function indexes between BYHW and control group after 3 months of treatment.

	BYHW (*n* = 34)	Control (*n* = 33)	*p* value
PT (s)			
Before treatment	12.18 ± 1.34	11.92 ± 1.55	0.473
After treatment	12.83 ± 2.39	12.00 ± 1.76	0.112
APTT (s)			
Before treatment	31.14 ± 4.14	30.99 ± 5.38	0.898
After treatment	34.30 ± 7.23*	32.30 ± 8.03	0.287
FIB (g/L)			
Before treatment	3.44 ± 0.75	3.43 ± 1.00	0.95
After treatment	2.83 ± 0.48**	3.08 ± 0.59*	0.054
TT (s)			
Before treatment	14.79 ± 2.37	14.87 ± 2.91	0.907
After treatment	16.28 ± 2.13**	15.07 ± 2.65	0.043

PT, prothrombin time; APTT, activation time of partial thromboplastin; FIB, fibrinogen; TT, thrombin time; compared with the same group before treatment, *p < 0.05, **p < 0.01.

### 3.4 Safety Evaluation

No significant difference in laboratory indicators between the BYHW and the control groups (*p* > 0.05; Please see Supplementary Material) was shown in the study. In addition, no significant ADRs were reported in the two groups during the trial. However, 4 patients died during the trial, all of whom died due to their disease deterioration unrelated to the investigational product. We recorded it in detail and made a report of it.

### 3.5 Proteomics Analysis

#### 3.5.1 Protein Quantification and Data Quality Control

**FIGURE 2:**
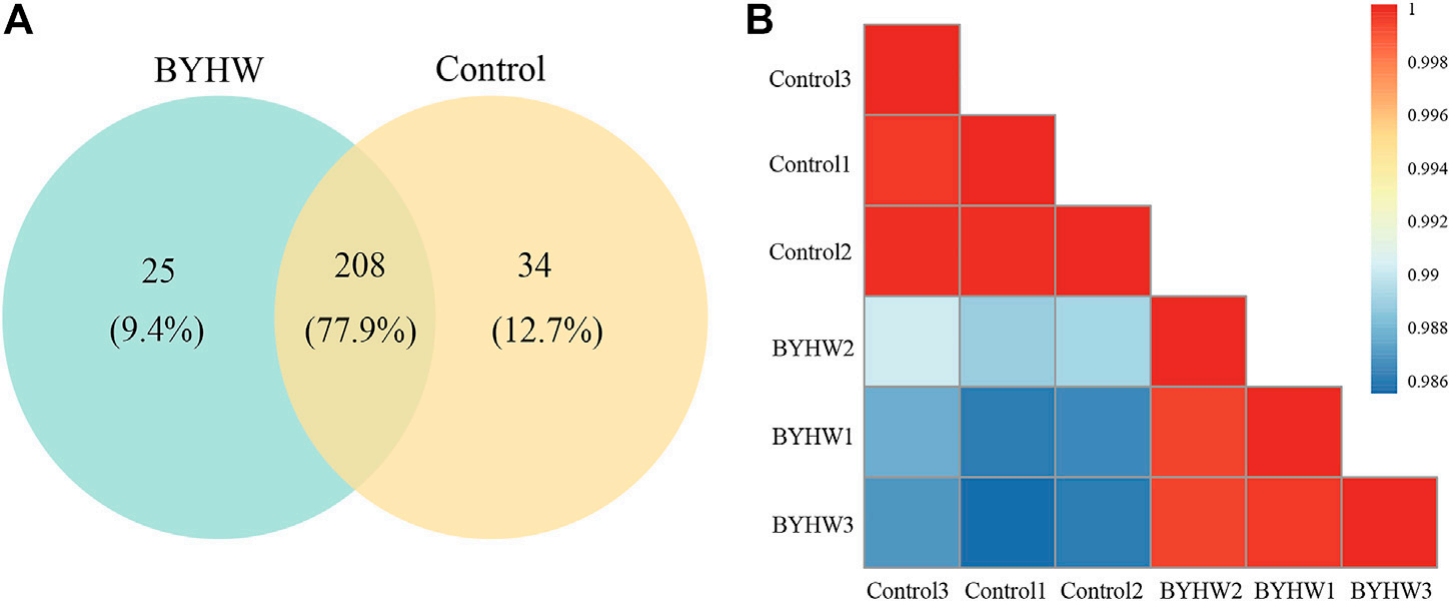
Statistical analysis of proteome data. **(A)** Venn diagram of BYHW and Control differential proteins; **(B)** Pearson correlation analysis.

Proteomics analysis displayed 267 non-redundant proteins from serum samples in the two groups. Among these proteins, there were a total of 208 proteins in both groups, accounting for 77.9% of the total proteins identified (see Figure 2A). The Pearson correlation between samples is shown in Figure 2B. The results show that the correlation coefficient between the two groups was low. The noticeable difference in the Pearson correlation coefficient within each group indicated that the samples between the two groups are relatively independent.

#### 3.5.2 Identification of Differentially Expressed Proteins

**FIGURE 3:**
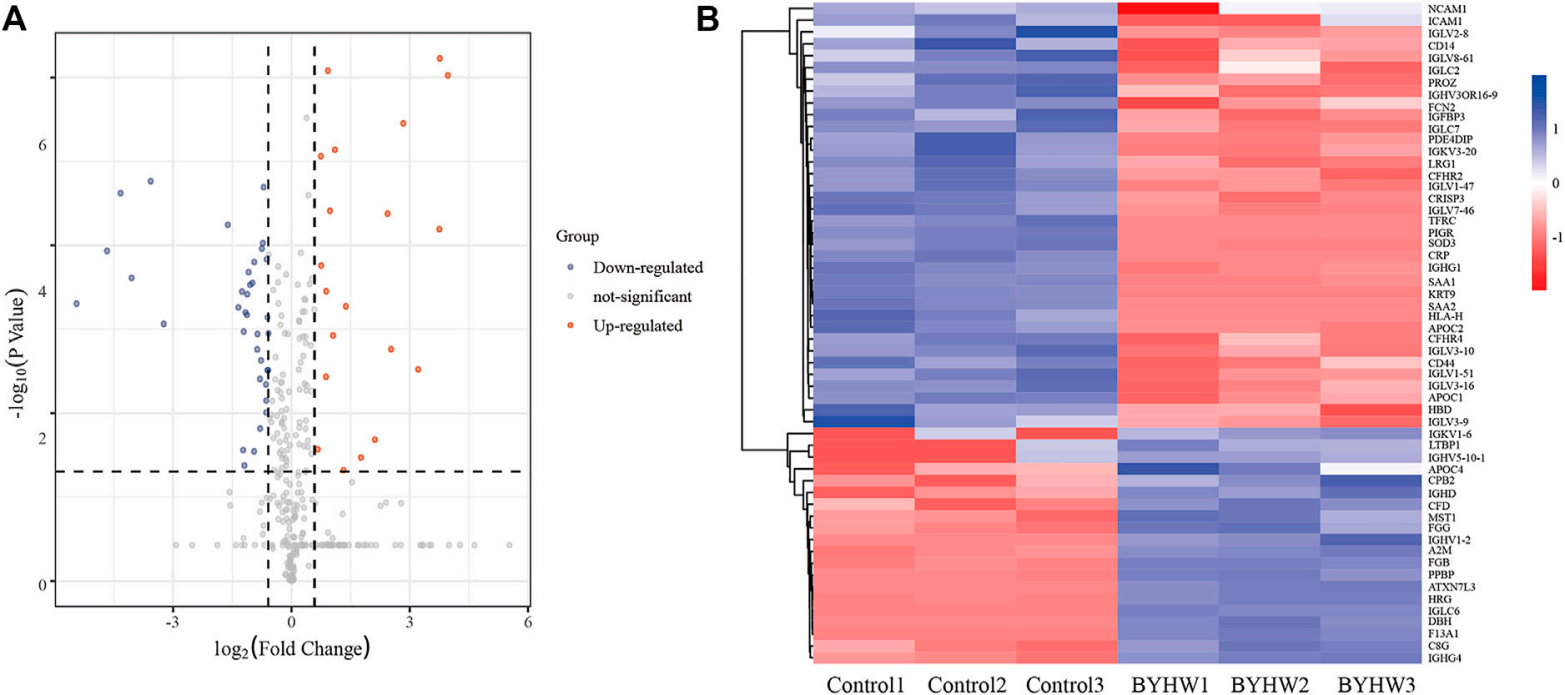
Integrated analysis of differential expression proteins. **(A)** The volcano map of BYHW/Control; **(B)** Heat map of 56 differentially expressed proteins.

In order to further explore the potential effect of BYHW on IHF, *p* < 0.05 (Student’s t-test) and folding change >1.5 were used as the analysis criteria. Compared with the control group, the study identified 56 differentially expressed proteins in the BYHW group, including 20 upregulated proteins and 36 downregulated proteins (Please see Supplementary Material). The volcanic map was further drawn according to the significance level and fold change value. In Figure 3A, the red dot represents the upregulated protein, the blue dot represents the downregulated protein, and the gray dot represents the non-differentially expressed gene. The heat map of cluster analysis showed that BYHW treatment caused notable changes in protein levels in the two groups (as shown in Figure 3B).

#### 3.5.3 Functional Classification of Differentially Expressed Proteins

**FIGURE 4:**
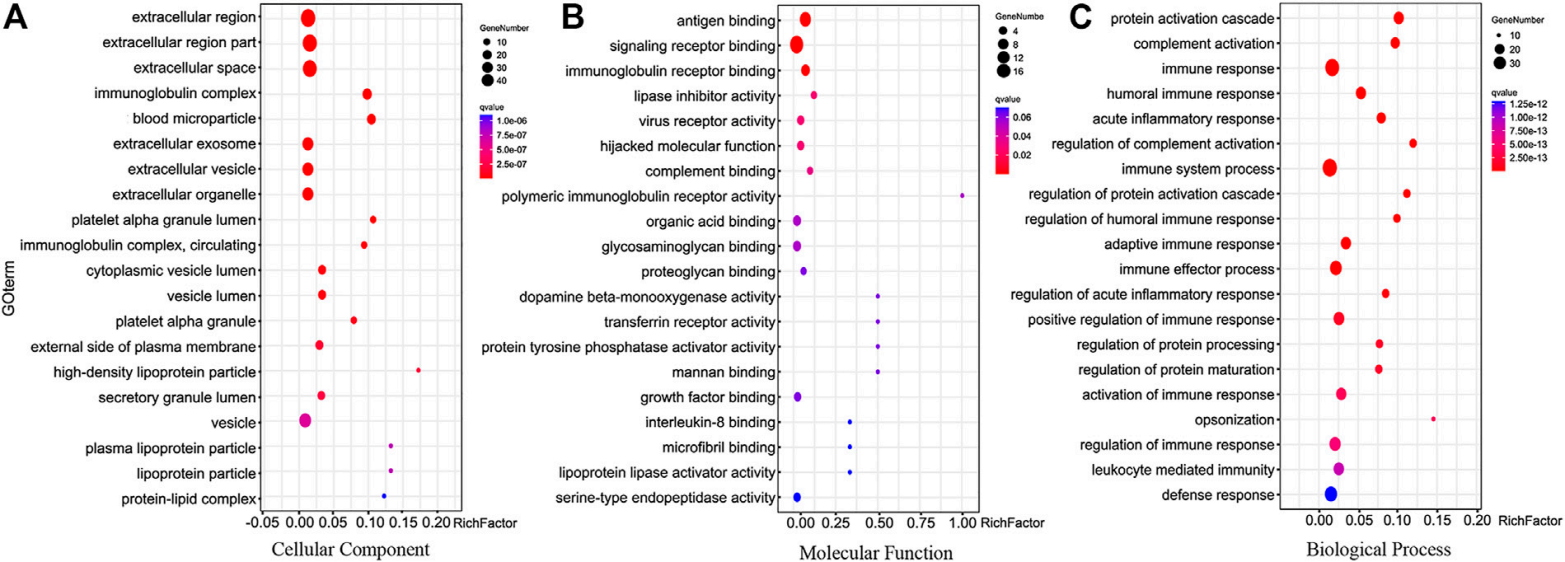
Biological function analysis of differentially expressed proteins. **(A)** Top 20 cellular component enrichment terms. **(B)** Top 20 molecular function enrichment terms. **(C)** Top 20 biological process enrichment terms.

GO analysis was performed using the OmicShare tools (https://www.omicshare.com/tools). GO covers three domains: biological process, molecular function, and cellular component, and the top 20 GO terms of each category were shown in Figure 4. The cellular component enrichment terms indicated that most of the differentially expressed proteins were located in the extracellular region, extracellular region part, extracellular space, immunoglobulin complex (Figure 4A). The analysis found that the molecular functions of the differentially expressed proteins mainly included antigen binding, signaling receptor binding, immunoglobulin receptor binding, lipase inhibitor activity (see Figure 4B). Biological processes mainly involved protein activation cascade, complement activation, immune response, humoral immune response (see Figure 4C).

#### 3.5.4 Biological Pathway Analysis of Differentially Expressed Proteins

**FIGURE 5:**
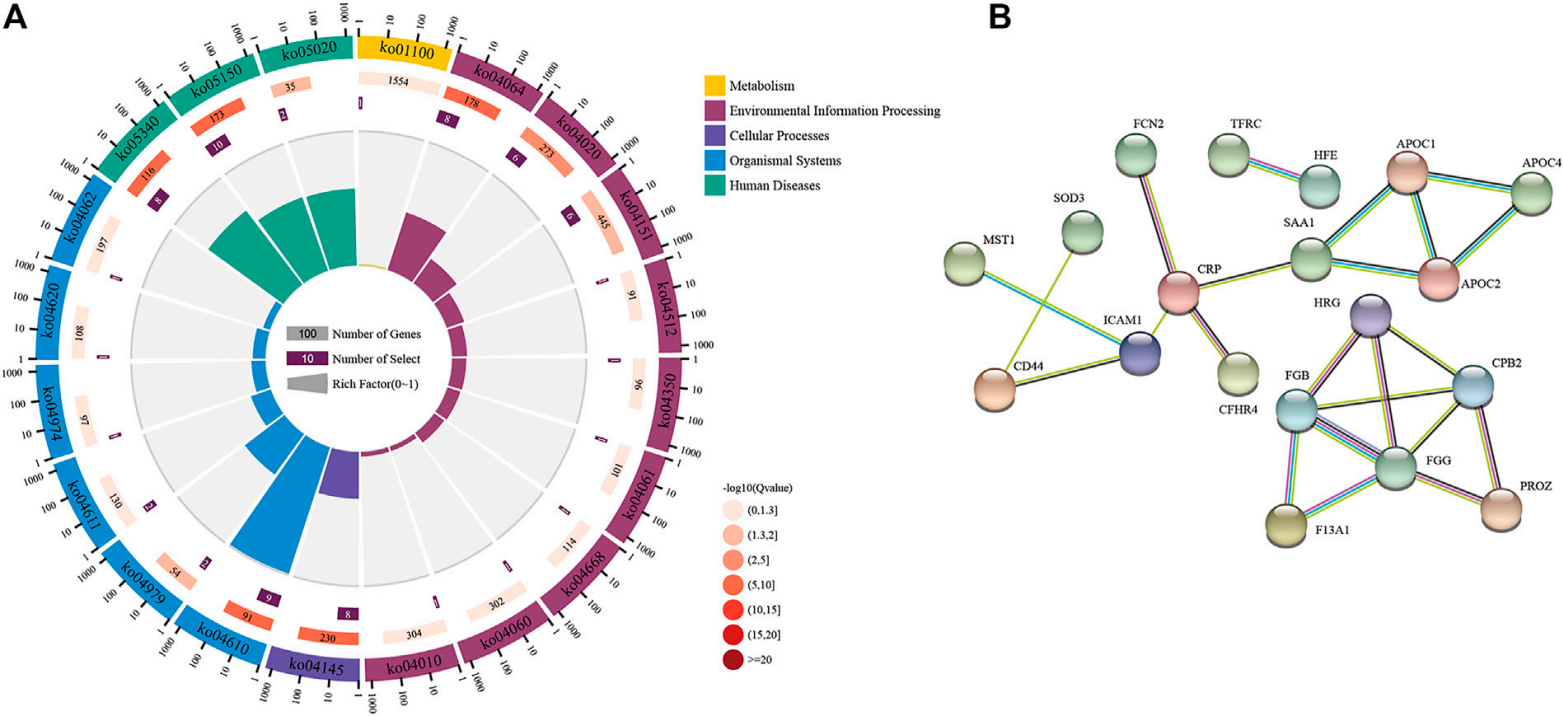
Pathway enrichment results and interaction relationship of different expressed proteins. **(A)** Top 20 biological pathway analysis of differentially expressed proteins; **(B)** Protein-protein interaction network differentially expressed proteins.

We performed a pathway enrichment analysis using the DAVID classification system (https://david.ncifcrf.gov/summary.jsp) and KEGG databases. The top 20 relevant pathways were shown in Figure 5A, and the coordinate ruler with the number of genes outside the circle; The second circle is the number of background genes and *p*-value. The more genes, the longer the bar will be. The redder the color, the smaller the *p*-value; The third circle is the total number of genes in each classification; The fourth circle represents the rich factor of each category (Qie et al., 2020). The differential proteins were associated with various biological pathways, including complement and coagulation cascades (ko04610), cholesterol metabolism (ko04979), NF-kappa B signaling pathway (ko04064), PI3K-Akt signaling pathway (ko04151), and metabolic pathways (ko01100).

#### 3.5.5 Protein-Protein Interaction Analysis of Differentially Expressed Proteins

The protein functions usually interact with each other to complete a series of biological processes in organisms. We thus applied the STRING database to predict all the protein interactions enriched in the pathway. Furthermore, we created a protein interaction network diagram to discover the critical nodes of BYHW treatment in the process of IHF. Set the highest confidence level, and the strongest connectivity included fibrinogen gamma (FGG), fibrinogen beta (FGB), Carboxypeptidase B2 (CPB2), Coagulation factor XIII A (F13A1), Intercellular adhesion molecule1 (ICAM1), Apolipoprotein C-II (APOC2), Apolipoprotein C-I (APOC1), CD44, suggesting that these proteins could be critical factors for BYHW intervention at the protein level (see Figure 5B).

## 4 Discussion

At present, modern medicine has reached a standstill for the pharmaceutical treatment of IHF. Based on the original effective neuroendocrine suppression, the role of drugs in further reducing the mortality and disability rate is more and more limited, while non-drug treatment is challenging to promote due to issues including technology, cost, and indication. Therefore, it is essential to develop new therapies to treat patients with IHF. BYHW was first published in *Yilin Gaicuo* by Wang Qingren, a famous doctor in the Qing Dynasty. It is a classical representative compound of supplementing qi and promoting blood circulation. According to the theory of “treating different diseases with the same therapy” in TCM, BYHW is applied to treat cardiovascular diseases and other diseases of qi deficiency and blood stasis syndrome with notable effects (Li et al., 2014).

In this study, we assessed the effectiveness and safety of BYHW in treating IHF for the first time through a randomized controlled trial with multiple endpoints. The combined results showed that BYHW significantly improved the clinical symptoms and cardiac function of IHF patients, ameliorated energy metabolism, and regulated coagulation function. We evaluated the clinical efficacy of BYHW in IHF by observing NYHA Classification, TCM syndrome scores, NT-proBNP, 6MWD, and LVEF. The results showed that BYHW significantly improved NYHA classification, TCM syndrome scores, and the percentage of subjects with at least 30% reduction in NT-ProBNP compared with the placebo; BYHW treatment significantly improved NT-ProBNP, 6 MWD, and LVEF compared with those before treatment. These findings suggested that BYHW, in combination with other standard therapies, may be a better cure for patients with IHF. As IHF is a complex syndrome, patients with IHF will display multiple symptoms, including dysregulation of the neuro-endocrine-immune network, energy metabolism disorders, and imbalance of coagulation mechanism (González et al., 2011). The results also showed that BYHW treatment significantly improved ADP/ATP Ratio, GLU, TC, LDL-C, APTT, FIB, and TT compared with those before treatment. We inferred that the therapeutic mechanism of BYHW may be related to the regulation of blood glucose and blood lipid levels, the improvement of energy metabolism and anticoagulant activity, and the reduction of the risk of thrombosis. Notably, almost patients included in the study tolerated well, and we tentatively assume that BYHW is relatively safe and does not increase the incidence of adverse effects. We also found that although most patients received guideline-guided heart failure therapy (ACEI/ARB, β-blockers, and Aldosterone receptor antagonist) at baseline, their proportion was lower than expected in global heart failure trials, which may reflect the gap in the actual treatment of patients with heart failure in most Asian countries.

In the present study, we investigated the underlying mechanism of BYHW on regulating serum proteome using label-free quantification. After screening and identification, we finally obtained 56 differentially expressed proteins, including 20 upregulated proteins and 36 downregulated proteins. The GO enrichment analysis revealed that these proteins were mainly located in the extracellular region, extracellular region part, extracellular space, and immunoglobulin complex. They participated in biological processes such as protein activation cascade, complement activation, immune response, and humoral immune response. KEGG pathway analysis showed that the pathways in which these differential proteins were mainly involved included complement and coagulation cascades, cholesterol metabolism, PI3K-Akt signaling pathway, NF-kappa B signaling pathway, and metabolic pathways.

The complement system is initially classified as a part of innate immunity. It is a strict self-regulatory system composed of the liquid phase, cell surface, and intracellular proteins. In the blood circulation, complements and coagulation cascades form a tight and complex network. They activate and regulate each other and jointly regulate immune monitoring and tissue homeostasis. Dysregulation of complements and coagulation cascades can lead to the progression of different clinical diseases (Luo et al., 2020). Other than our research, multiple studies have revealed that Chinese medicine can play a role in treating cardiovascular diseases through complement and coagulation cascades (Wang P et al., 2020; Wang, J et al., 2020). Atherosclerosis (AS) is one of the leading causes of cardiovascular disease. Research has shown that Cholesterol metabolism and dyslipidemia are involved in the occurrence of AS (Ke and Shen, 2019), which may have a direct or indirect adverse effect on the long-term prognosis of myocardial infarction. The reason may be the degree of ischemic injury, or the subsequent events (e.g., recurrent myocardial infarction) increase the risk of HF (Gerber et al., 2016). PI3K/Akt signaling pathway plays an essential role in angiogenesis. In other words, PI3K acts as a crucial signal for regulating cell metabolism, proliferation, and apoptosis. It also plays a vital role in the activation of the Akt-dependent signaling pathway. A variety of vasoactive factors can be controlled by Akt activation (Lin et al., 2015). The PI3K/Akt signaling pathway regulates inhibits oxidative stress, improves cardiac dysfunction and hemodynamics, and alleviates myocardial fibrosis (Zhong et al., 2020). NF-kappa B is an essential nuclear transcription factor involved in regulating cell differentiation and apoptosis in organisms and controlling inflammatory and immune responses. Activation of the NF-kappa B signaling pathway may be associated with aggravation of heart failure (Li et al., 2018). Previous studies have also shown that BYHW can inhibit NF-kappa B signaling pathway and adjust blood lipid levels to treat AS (Liu et al., 2020).

Based on the observation of the protein-protein interaction functional network, we discovered that FGG, FGB, CPB2, F13A1, ICAM1, APOC2, APOC1, and CD44 were located in the center of the network, serving as a hub to interact with other proteins. The proteins mainly participated in a variety of biological processes, including coagulation systems and energy metabolism. Moreover, fibrinogen level plays an essential role in coagulation, hemostasis, and inflammation, a recognized risk factor for cardiovascular diseases. Fibrinogen circulates as a dimer in the plasma, consisting of three pairs of polypeptide chains alpha, beta, and gamma encoded by fibrinogen alpha (FGA), FGB, and FGG genes (Simurda et al., 2020). In addition, fibrinogen acts as a crucial agent in treating IHF: it can promote platelet aggregation, promote the growth, proliferation, and contraction of smooth muscle and endothelial cells. It also increase blood viscosity and peripheral resistance, causing endothelial cell damage. More importantly, fibrinogen promotes red blood cell adhesion and thrombosis, which are crucial for the occurrence of cardiovascular diseases (Kunutsor et al., 2016). Previous studies have also shown that TCM could regulate FGA and FGG levels to improve coagulation function and play a role in treating coronary heart disease (Wang, J et al., 2020). As for CPB2, it inactivates a variety of inflammatory mediators by removing c-terminal arginine. It also removes c-terminal lysine from partially degraded fibrin, thereby reducing tissue plasminogen activator and plasminogen binding to clots, eventually reducing plasmin formation (Claesen et al., 2021). Evidence showed that the CPB2 level in the thrombus site in patients with acute myocardial infarction is exceptionally high, and it is helpful to the thrombus dynamics of plaque rupture site (Leenaerts et al., 2015). Additionally, we discovered that BYHW could upregulate CPB2 by proteomics, which plays a beneficial role in treating IHF. The F13A1 protein found in BYHW affects many physiological processes and plays a crucial role in balancing thrombus formation and dissolution. Pharmacological studies have shown that F13A1 may be a promising drug target for developing new anticoagulants with limited bleeding risk (Al-Horani and Kar, 2020). As for ICAM1, other studies defined the protein’s crucial role in the development of heart failure by participating in the process of inflammation and apoptosis (Patel et al., 2020). ICAM1 has the potential as a biomarker and therapeutic target for post-acute myocardial infarction heart failure (Lino et al., 2019). Numerous drugs for heart failure can inhibit the expression of ICAM and have anti-inflammatory effects (Krychtiuk et al., 2015), which matched with the results of our study. Based on previous studies, many apolipoproteins seem involved in ventricular remodeling and HF worsening (Jang et al., 2020). APOC2 is a component of chylomicrons, very LDL, LDL, and HDL. The existing evidence supports the significant correlation between APOC2 and coronary artery stenosis (Ku et al., 2020). APOC1 is directly related to cardiovascular physiology by controlling plasma lipid levels (Fuior and Gafencu, 2019). In addition, APOC1 is the only known endogenous cholesteryl ester transfer protein inhibitor, and this constitutive effect of APOC1 is impaired in coronary artery disease with dyslipidemia (Sacks et al., 2020). Likewise, our study showed that BYHW may regulate APOC1 and APOC2, further improving energy metabolism and playing a therapeutic role. CD44 is a widely expressed transmembrane glycoprotein involved in various cell functions, including cell adhesion, migration, proliferation, and differentiation. The defect of the CD44 signal cascade may play an essential role in the pathogenesis of adverse remodeling after myocardial infarction (Frangogiannis, 2019). In addition, CD44, hyaluronan, and their interactions are indispensable in developing myocardial fibrosis and cardiac remodeling after myocardial infarction, accelerating the progress of heart failure (Suleiman et al., 2018). These core proteins could be closely related to the development of IHF, and BYHW could play a therapeutic role in IHF through these targets.

Our study has some limitations. To begin with, we only detected the serum proteome of patients after treatment and need to further examine the serum proteome changes at other time points. In addition, the chemical composition of TCM is complex, and the practical components of BYHW that play a therapeutic role deserve to be further studied.

## 5 Conclusion

In conclusion, BYHW could further improve cardiac dysfunction and clinical symptoms in patients with IHF, ameliorate energy metabolism and regulate coagulation function in the context of standard medical treatment for IHF. We also demonstrated the safety of BYHW in clinical application. Proteomics analysis showed that BYHW may exert therapeutic effects on IHF by improving energy metabolism and regulating coagulation function through multiple targets (FGG, FGB, CPB2, F13A1, ICAM1, APOC2, APOC1, and CD44) and pathways (complement and coagulation cascades, cholesterol metabolism, NF-kappa B signaling pathway, PI3K-Akt signaling pathway, and metabolic pathways). This study greatly expands our knowledge, provides scientific support for the potential therapeutic mechanism of BYHW in treating IHF, and offers a possible alternative for the prevention and treatment of IHF.

## Data Availability

The datasets presented in this study can be found in online repositories. The name of the repository and accession number can be found below: ProteomeXchange; PXD029771.

## Abbreviations

6MWD, 6-minute walk distance; ATP, adenosine triphosphate; ADP, adenosine diphosphate; APOC1, Apolipoprotein C-I; APOC2, Apolipoprotein C-II; APTT, activation time of partial thromboplastin; BYHW, Buyang Huanwu Decoction; CPB2, Carboxypeptidase B2; F13A1, Coagulation factor XIII A; FGB, fibrinogen beta; FGG, fibrinogen gamma; FIB, fibrinogen; GLU, glucose; HDL-C, high density lipoprotein cholesterol; ICAM1, intercellular adhesion molecule1; IHF, ischemic heart failure; LDL-C, low density lipoprotein cholesterol; LVEF, left ventricular ejection fraction; NT-ProBNP, N-terminal pro-B-type natriuretic peptide; NYHA, New York Heart Association; PT, prothrombin time; TC, total cholesterol; TCM, traditional Chinese medicine; TG, triglyceride; TT, thrombin time.
