# Supplementary material for: Efficacy and Mechanism of Buyang Huanwu Decoction in Patients With Ischemic Heart Failure: A Randomized, Double-Blind, Placebo-Controlled Trial Combined With Proteomic Analysis
Source: Front Pharmacol. 2022 Mar 18;13:831208. doi: 10.3389/fphar.2022.831208 (PMC8971676; doi:10.3389/fphar.2022.831208)
Supplement: Supplementary file 1 [file DataSheet1.docx]

Supplementary Material

**The characteristic map and chemical composition of Buyang Huanwu Decoction (BYHW)**

The characteristic map and chemical composition of the BYHW were shown in Figure 1 and Table 1. A variety of active chemical components can be detected in BYHW, such as Ligustrazine, oxypaeoniflora, chlorogenic acid, albiflorin, paeoniflorin, calycosin, 7-o-glucoside, rutin, ferulic acid, Senkyunolide I, Senkyunolide H, Formononetin.


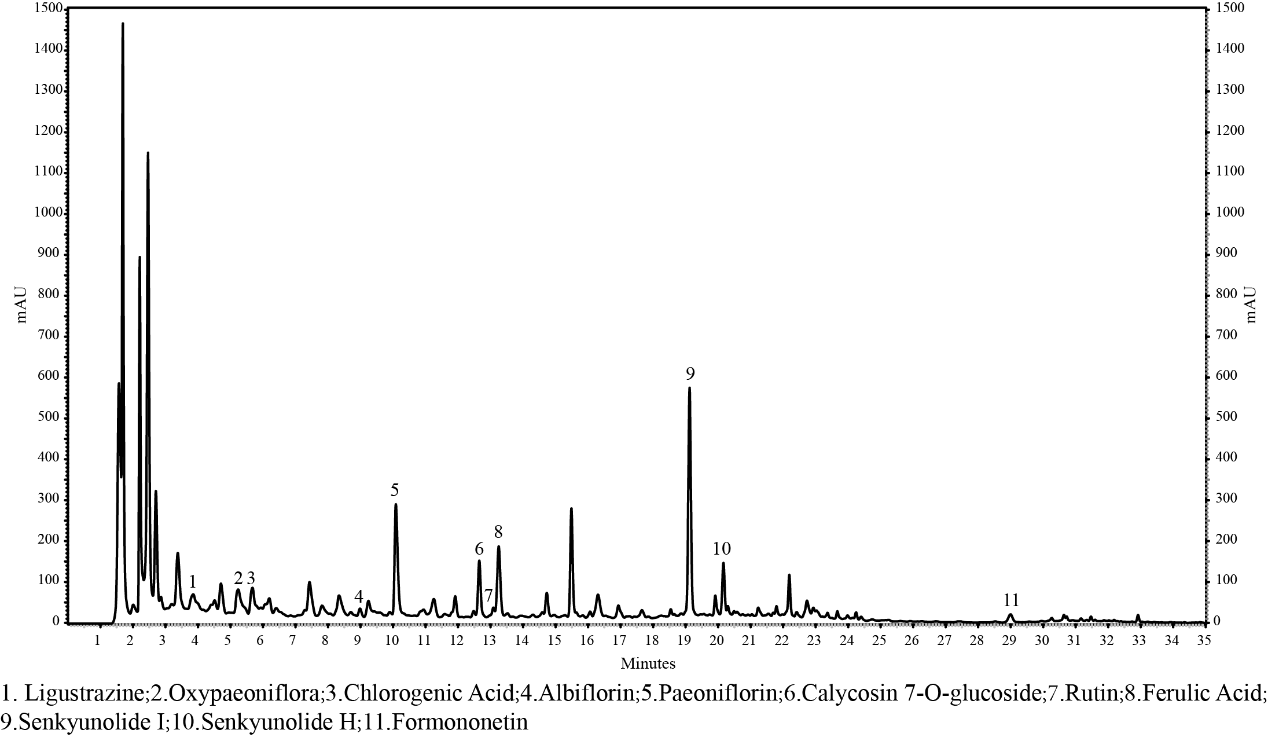


**Figure 1.** Chemical fingerprint of BYHW. Experimental conditions: Agilent RRHD SB C18 (3.0×150 mm,1.8 μm); 0.1% phosphoric acid solution and acetonitrile; 254 nm; Column temperature 35℃; 0.4 ml/min; 2 μL.

Table 1. Identification results of chemical components

| No. | Compound | Maximum absorption wavelength (nm) | Relative retention time (min) |
| --- | --- | --- | --- |
| 1 | Ligustrazine | 302 | 3.72 |
| 2 | Oxypaeoniflora | 258 | 5.08 |
| 3 | Chlorogenic Acid | 327 | 5.52 |
| 4 | Albiflorin | 235 | 8.793 |
| 5 | Paeoniflorin | 235 | 9.913 |
| 6 | Calycosin 7-O-glucoside | 255 | 12.433 |
| 7 | Rutin | 355 | 12.84 |
| 8 | Ferulic Acid | 323 | 13.033 |
| 9 | Senkyunolide I | 275 | 18.813 |
| 10 | Senkyunolide H | 275 | 19.827 |
| 11 | Formononetin | 255 | 28.66 |

**1. Method: LC-MS/MS method**

**2. Instruments and Materials**

Ultrahigh performance liquid chromatography (Agilent Technology 1290 infinity), automatic sampler, column temperature box, DAD detector and online analysis software. Ultrasonic cleaner (Jining Hengtong Electronic Equipment Co., Ltd., HT-300BQ), electronic balance (METLLER TOLEPO, MS105DU/AL204101), filter (JINTENG Co., Ltd., NO.0951064, 0.22μm), microporous membrane (JINTENG Co., Ltd., NO.0647037, 0.22μm).

**3. Main reagents**

Methanol (chromatography pure, Fisher,167921);

Acetonitrile (chromatography pure, Fisher,170236);

Phosphoric acid (chromatography pure, Fisher,158625);

Pure water (China Hangzhou Wahaha Co., Ltd).

Reference substance : Calycosin isoflavone glucoside ( 111920-201505,97.1 % ), Astragaloside IV(110781-201616, 97.4%), Formononetin (111703-201504, identification), Quercetin (100081-201408, 99.1%), Kaempferol (110861-201310, 93.2%), Hydroxysafflor Yellow A (111637-201308, 96.5%), Gallic acid (110831-201605, 90.8%), Paeonol (110708-201407, 99.9%) were purchased from China Food and Drug Verification Institute; Rutin (153-18-4, 94.1%), Catechin (154-23-4, 100%), Benzoyl paeoniflorin (38642-49-8, 96.4%) were purchased from Shanghai Shidande Biotechnology Co., Ltd; Astragaloside I (84680-75-1, 99.43%), Ligustrazine (1124 - 11 - 4, 98.49%), Ferulic acid (1135 - 24 - 6, 98.75%), Z-Ligustilide (G-5601, 98.64%), Senkyunolide H (94596-27-7, 98.71%), Senkyunolide I (94596 -28-8, 98.71%) were purchased from Stander Chemical Technology Co., Ltd., Shenzhen; Chlorogenic acid (FY14351025, 99.74%), Amygdalin (FY13871222, 97.09%), Paeoniflorin (FY15890529,98.26%), Albiflorin (FY15930319, 99.01%), Oxypaeoniflora (FY17500119, 99.55%), 3-Butylidenephthalide (FY20541015, 96.6%), 3-*n*-Butylphthalide (FY20550407, 98.64%) was purchased from Nantong Feiyu Biotechnology Co., Ltd. BYHW granule (Sanjiu Medical & Pharmaceutical Co., Ltd., 1511001Y).

**4. Preparation of reference solution**

Accurately weigh the reference substance Calycosin isoflavone glucoside, Astragaloside IV, Formononetin, Quercetin, Kaempferol, Hydroxysafflor Yellow A, Gallic acid, Paeonol, Rutin, Catechin, Benzoyl paeoniflorin, Astragaloside I, Ligustrazine, Ferulic acid, Z-Ligustilide, Senkyunolide H, Senkyunolide I, Chlorogenic acid, Amygdalin, Paeoniflorin, Albiflorin, Oxypaeoniflora, 3-Butylidenephthalide, 3-n-Butylphthalide, each 10 mg in 10 mL volumetric flask, with methanol to the calibration line, namely 1 mg / mL of the reserve solution. A suitable amount of reserve solution was diluted to 200 μg / mL reference solution, and 0.22 μm microporous membrane was used for filtration.

**5. Preparation of test solution**

Take 4 g of ground sample powder, accurately weigh it, put it into 50ml centrifuge tube, add 20ml of 80% methanol solution to dissolve it, ultrasonic treatment (power 300W, 40KHz) for 30min, cool it to room temperature, shake it up, filter with 0.22 μm microporous membrane and get it.

**6. chromatographic condition**

Chromatographic column: Agilent RRHD SB C18 (3.0 × 150 mm, 1.8 μm); mobile phase A: 0.1 % phosphoric acid solution; mobile phase B: acetonitrile. Elution by gradient in Table 2; detection wavelength: 254 nm; column temperature: 35 °C, flow rate: 0.4 mL / min; injection volume 2μL.

**Table 2.** Liquid chromatography elution gradient

| Time (min) | A（%） | B（%） |
| --- | --- | --- |
| 0 | 90 | 10 |
| 15 | 79 | 21 |
| 21 | 65 | 35 |
| 27 | 65 | 35 |
| 30 | 40 | 60 |
| 35 | 30 | 70 |
| 40 | 10 | 90 |
| 40.1 | 90 | 10 |
| 45 | 90 | 10 |

**Safety evaluation**

**Table 3.** Safety evaluation: comparison of incidence of abnormal laboratory indexes between BYHW and control groups.

| Laboratory index | BYHW (n = 34) | Control (n = 33) |
| --- | --- | --- |
| Blood routine |  |  |
| White blood cell count | 1(2.94%) | 0(0.00%) |
| Red blood cell count | 2(5.88%) | 1(3.03%) |
| Hemoglobin | 3(8.82%) | 3(9.09%) |
| Blood platelet count | 2(5.88%) | 1(3.03%) |
| Urine routine |  |  |
| Urine erythrocyte | 2(5.88%) | 1(3.03%) |
| Urine white blood cell count | 2(5.88%) | 4(12.12%) |
| Urine protein | 1(2.94%) | 2(6.06%) |
| Hepatic and renal function |  |  |
| Alanine transaminase (ALT) | 2(5.88%) | 3(9.09%) |
| Glutamic oxalacetic transaminase (AST) | 3(8.82%) | 3(9.09%) |
| Urea nitrogen | 4(11.76%) | 3(9.09%) |
| Creatinine | 2(5.88%) | 2(6.06%) |
| Electrolytes |  |  |
| K | 0(0.00%) | 0(0.00%) |
| Na | 0(0.00%) | 1(3.03%) |
| Cl | 0(0.00%) | 0(0.00%) |

**Identification of differentially expressed proteins**

**Table 4.** The differentially expressed proteins of BYHW/Control

| No. | Accession | Gene name | Fold change | Tendency | No. | Accession | Gene name | Fold change | Tendency |
| --- | --- | --- | --- | --- | --- | --- | --- | --- | --- |
| 1 | P01893 | HLA-H | 0.46 | down | 29 | A0A075B6I9 | IGLV7-46 | 0.66 | down |
| 2 | P02786 | TFRC | 0.11 | down | 30 | A0A075B6K5 | IGLV3-9 | 0.59 | down |
| 3 | P02741 | CRP | 0.06 | down | 31 | P01701 | IGLV1-51 | 0.58 | down |
| 4 | P35527 | KRT9 | 0.05 | down | 32 | A0A075B6K4 | IGLV3-10 | 0.45 | down |
| 5 | P01833 | PIGR | 0.04 | down | 33 | P02654 | APOC1 | 0.42 | down |
| 6 | P0DJI9 | SAA2 | 0.02 | down | 34 | P02750 | LRG1 | 0.52 | down |
| 7 | P05362 | ICAM1 | 0.43 | down | 35 | P02655 | APOC2 | 0.66 | down |
| 8 | Q15485 | FCN2 | 0.43 | down | 36 | P01857 | IGHG1 | 0.47 | down |
| 9 | P13591 | NCAM1 | 0.44 | down | 37 | Q14766 | LTBP1 | 0.61 | up |
| 10 | P22891 | PROZ | 0.57 | down | 38 | P09172 | DBH | 2.50 | up |
| 11 | P08294 | SOD3 | 0.33 | down | 39 | P00488 | F13A1 | 5.41 | up |
| 12 | Q92496 | CFHR4 | 0.39 | down | 40 | P23083 | IGHV1-2 | 7.13 | up |
| 13 | A0A075B6I0 | IGLV8-61 | 0.64 | down | 41 | P55056 | APOC4 | 5.76 | up |
| 14 | P36980 | CFHR2 | 0.50 | down | 42 | A0A0C4DH72 | IGKV1-6 | 1.59 | up |
| 15 | P0DOY2 | IGLC2 | 0.64 | down | 43 | Q14CW9 | ATXN7L3 | 3.39 | up |
| 16 | P01709 | IGLV2-8 | 0.52 | down | 44 | P0CF74 | IGLC6 | 13.46 | up |
| 17 | P02042 | HBD | 0.66 | down | 45 | P00746 | CFD | 15.62 | up |
| 18 | A0A075B6K0 | IGLV3-16 | 0.46 | down | 46 | A0A0J9YXX1 | IGHV5-10-1 | 1.84 | up |
| 19 | P01700 | IGLV1-47 | 0.60 | down | 47 | Q96IY4 | CPB2 | 4.33 | up |
| 20 | P16070 | CD44 | 0.67 | down | 48 | P26927 | MST1 | 1.83 | up |
| 21 | P54108 | CRISP3 | 0.65 | down | 49 | P02679 | FGG | 2.08 | up |
| 22 | P0DJI8 | SAA1 | 0.08 | down | 50 | P02775 | PPBP | 9.27 | up |
| 23 | Q5VU43 | PDE4DIP | 0.55 | down | 51 | P02675 | FGB | 2.15 | up |
| 24 | P17936 | IGFBP3 | 0.59 | down | 52 | P01880 | IGHD | 13.56 | up |
| 25 | P08571 | CD14 | 0.64 | down | 53 | P07360 | C8G | 2.60 | up |
| 26 | P01619 | IGKV3-20 | 0.55 | down | 54 | P04196 | HRG | 1.69 | up |
| 27 | A0M8Q6 | IGLC7 | 0.48 | down | 55 | P01861 | IGHG4 | 1.90 | up |
| 28 | S4R460 | IGHV3OR16-9 | 0.46 | down | 56 | P01023 | A2M | 1.97 | up |
